# Hierarchical biomechanical characterisation of riboflavin-UVA crosslinking and decorin treatment in the porcine cornea

**DOI:** 10.3389/fbioe.2025.1603679

**Published:** 2025-09-24

**Authors:** J. S. Bell, S. R. Morgan, O. Shebanova, S. L. Evans, C. Boote, N. Terrill, K. M. Meek, S. Hayes

**Affiliations:** ^1^ School of Physics and Astronomy, Cardiff University, Cardiff, United Kingdom; ^2^ School of Optometry and Vision Sciences, Cardiff University, Cardiff, United Kingdom; ^3^ Diamond Light Source Ltd, Didcot, United Kingdom; ^4^ School of Engineering, Cardiff University, Cardiff, United Kingdom

**Keywords:** riboflavin/UVA crosslinking, decorin, cornea, biomechanics, synchrotron X-ray scattering, collagen, crimp

## Abstract

**Introduction:**

The mechanisms underpinning the stiffening and stabilising effect of riboflavin/UVA crosslinking on the corneal stroma are not well understood. We report the findings of a biomechanics and synchrotron X-ray scattering study aimed at quantifying hierarchical strain mechanisms in treated and untreated porcine corneas. We applied the same approach to specimens treated with human recombinant decorin core protein, in isolation and in conjunction with riboflavin/UVA.

**Methods:**

Tensile testing was carried out in conjunction with simultaneous synchrotron X-ray scattering. Diffraction peaks associated with the interfibrillar spacing and D-period of collagen were fit to bespoke models to quantify fibril elongation and reorientation under load.

**Results:**

Riboflavin/UVA crosslinking stiffened corneas by approximately 60% while decorin treatment did not significantly affect the mechanical properties. Correlations between fibril elongation caused by applied tensile strain and bulk stiffness were used to approximate fibril stiffness, values for which were relatively similar for control and treatment groups, compared with the magnitude of difference in the bulk stiffness alone.

**Discussion:**

The results imply the bulk stiffening caused by crosslinking was not primarily due to increases in fibril stiffness. Instead, trends in bulk fibril reorientation and straightening/uncrimping imply the stiffening is attributable to enhanced interconnectivity of the fibrillar stroma, leading to greater fibril recruitment fraction. The techniques reported here are applicable to a wide range of tissues for the evaluation of new, existing and adjuvant therapies.

## Introduction

The human cornea is the primary lens of the eye and thus has to fulfil the criteria of being transparent, precisely curved and sufficiently tough to protect the eye from insult. It achieves these criteria primarily through the precise hierarchical arrangement of fibrillar collagen. Keratoconus is a degenerative disease of the cornea that disrupts the organisation of collagen (Koster et al., 2013), causing weakening and changes in macroscopic curvature, which has a severely detrimental impact on patient quality of life and can cause blindness (Kymes et al., 2008).

Riboflavin/UVA cross-linking therapy, commonly referred to as “CXL”, was first introduced in the early 2000s as a corneal stiffening therapy for halting keratoconus progression (Wollensak et al., 2003a). Since then, the long-term effectiveness of the treatment for stabilising the cornea has been demonstrated in numerous clinical trials, with follow-ups of up to 10 years (Raiskup et al., 2015) CXL therapy involves soaking the cornea with a photosensitiser, riboflavin (vitamin B2) and subsequently exposing it to UVA light (365 nm) with a radiant exposure of 5.4 mJ/cm. The resulting photochemical process leads to the production of oxygen radicals within the corneal stroma and the creation of covalent connections between amino acids, referred to as “crosslinks”. Ultrastructural and biochemical analyses have indicated that the crosslinks formed during CXL therapy, which increase the stiffness (Wollensak et al., 2003b; Hammer et al., 2014) and enzymatic resistance (Aldahlawi et al., 2015; Spoerl et al., 2004) of the cornea, likely occur between collagen molecules at the surface of the fibrils and within the proteoglycan-rich coating surrounding them (Hayes et al., 2013; Zhang et al., 2011). However, the fundamental mechanisms underpinning the enhancement of the cornea’s biomechanical properties remain elusive, and so the potential for manipulating tissue stiffening effects for adjuvant therapies remains unknown.

Interestingly, it has been reported that a 60 s application of human recombinant decorin core protein to de-epithelialised *ex vivo* corneas leads to an immediate tissue stiffening effect that is comparable in magnitude to that achieved with CXL (Pappa et al., 2021; Metzler et al., 2016). Decorin is a well characterised, small leucine rich proteoglycan that is naturally expressed in relatively high levels throughout the corneal stroma at all stages of development (Zhang et al., 2009). Its 40 kDa core protein is a horseshoe-shaped protein (Scott, 1996) that binds to Type I collagen fibrils at specific sites, namely, the ‘d’ and ‘e’ bands, with its associated covalently bound glycosaminoglycan side chains spanning the interfibrillar spaces (Scott and Haigh, 1988; Meek et al., 1986). Molecular modelling suggests that each horseshoe-shaped decorin core protein monomer binds to the collagen fibril surface through interactions with at least four separate collagen molecules from four individual collagen microfibrils, thereby forming bridges that help to stabilise the fibrils (Orgel et al., 2009). We postulate that application of exogenous human recombinant decorin core protein to the cornea, in conjunction with CXL, could lead to an enhanced crosslinking procedure with greater achievable tissue stiffening as their combined actions may further stabilise corneal collagen fibrils and the proteoglycan-rich coating surrounding them.

Fibrillar collagen is known for its remarkable tensile stiffness and strength, which is often compared with that of steel, however this belies its behaviour in non-weightbearing tissues such as the cornea, where its compliance is equally remarkable. Corneal collagen differs from that of tendon and other high load-absorbing tissues by exhibiting a significant supramolecular twist (Reale et al., 1981; Ottani et al., 2002; Ottani et al., 2001; Holmes et al., 2001), meaning tropocollagen molecules are coiled within their constituent fibrils. This architecture endows corneal collagen fibrils (and that of other non-loadbearing tissues such as skin) with the “spring-like” ability to elongate under very small loads–loads of physiological magnitude in the cornea (Bell et al., 2022; Bell et al., 2018). The hierarchical mechanisms by which corneal collagen accommodates strain can be quantified using X-ray scattering (Aghamohammadzadeh et al., 2004; Worthington and Inouye, 1985), due to the quasi-crystalline arrangement of fibrils. Tissue-scale strains in the physiological range, such as those caused by blinking, eye movement or intraocular pressure fluctuations, are typically attributed to straightening of fibril crimp (Grytz and Meschke, 2009) and, more recently, fibril elongation (Bell et al., 2022) (crimp is the wavy arrangement of fibrils that can be straightened under load. The extent to which a crimped fibril can be extended by fully straightening it without stretching is usually referred to as its tortuosity). It is worth noting that a significant proportion of corneal collagen remains crimped at physiological intraocular pressure (Jan et al., 2018). Changes in the macroscopic mechanical properties of the cornea, such as those caused by CXL treatment, should therefore manifest as some change in either or both of these strain mechanisms.

In this study we aim to develop a mechanistic understanding of CXL and decorin treatment by using synchronized biomechanical testing and X-ray scattering to quantify changes in corneal collagen architecture in response to tensile load. This approach allows stiffness to be directly correlated with variations in hierarchical strain mechanisms, allowing a picture to be built of how crosslinking may stiffen the cornea and halt the progression of keratoconus. In addition to this, we explore the tissue stiffening effects of exogenous human recombinant Decorin core protein when applied in isolation and in combination with CXL to assess the therapeutic potential of these treatments.

## Methods

### Specimen preparation

46 porcine eyes were obtained and transported on ice from a local DEFRA licensed abattoir within 6 h of euthanasia and divided into the treatment groups described below. In all cases, the corneal epithelium was removed prior to treatment. In Groups 1 and 2 (Decorin; n = 10 and Dec + CXL; n = 7), an 11-mm diameter corneal well was used to apply 300 µL human recombinant decorin core protein (Galacorin^®^) in a phosphate buffered saline solution (5 mg/mL Galacorin^®^ at pH 7.2 supplied by Phoenicis Therapeutics, Massachusetts, USA). After 60s the corneal well was removed, and the solution was gently wiped away from the corneal surface. In the Dec + CXL group, the decorin treatment was immediately followed by riboflavin/UVA cross-linking, involving a 16-min application of 0.1% riboflavin/1.1% HPMC solution (Mediocross M) to the stromal surface followed by UVA exposure (365 nm UVA light with a fluence of 9 mW/cm^2^, exposure time of 10 min and an 11-mm beam diameter). In Group 3 (CXL, n = 10), eyes underwent the riboflavin/UVA cross-linking procedure without a decorin pre-treatment. The remaining eyes served as either riboflavin-only controls (Ribo, n = 10), receiving a 20-min application of 0.1% riboflavin/1.1% HPMC solution (Mediocross M) to the stromal surface without UVA exposure, or as untreated controls (n = 9). The central corneal thickness of each eye was measured using a SP-100 portable pachymeter (Tomey GmbH Technology and Vision, Nurnberg, Germany) before and after each treatment. All samples were wrapped in plastic film (to maintain tissue hydration) and stored at 4 °C overnight until required for data collection.

Untreated specimens were used solely for the purpose of testing equipment and internal controls. The non-irradiated, riboflavin-soaked corneas were used as controls for the treatment groups to minimise the effect of hydration differences on the biomechanical and structural data. Mechanical and equipment failure meant full datasets were not acquired from every specimen. 9 Ribo, 8 CXL, 8 Dec and 6 Dec + CXL datasets were obtained in total.

### Mechanical testing

Specimens were tested approximately 24 h following treatment. Central corneal thickness was measured and then a strip approximately 3.5 mm in width was excised using a custom-designed punch comprising two precisely aligned parallel razor blades. In all cases, the strip was obtained in the superior-inferior direction.

A custom-built tensile tester comprising a piezo linear stage (Q-545-240, PI, driven by PIShift E−871 controller) and 4.9 N tension/compression load cell (Model 31, RDP) was used to apply loads to the tensile strips. Specimens were attached to micromanipulator arms using cyanoacrylate glue and a tare load was applied to straighten them. This straightened state was used as the reference configuration for each specimen and subsequent measurements and analyses were based on this consistent baseline, ensuring internal validity across specimens. During preparation and while waiting for the glue to cure, the specimens were sprayed periodically with distilled water using a perfume mister, taking care not to wet the glue. Distilled water was used to replace water lost due to air drying in the low-humidity environment of the synchrotron (in this instance PBS or similar osmotically-balanced solutions are inappropriate as they add additional solute, which can affect hierarchical structure and mechanics (Huang and Meek, 1999)). The specimens were then strained to 8% at a constant rate over 3 min, during which time X-ray scatter patterns were acquired from the centre. Strains of interest were 2% (IOP-induced hoop stress induces strains of approximately 2%), 5% and 8% (for comparison with other tensile studies of cornea (Hatami-Marbini and Jayaram, 2018; Hatami-Marbini and Rahimi, 2015; Boyce et al., 2007)). Note that gross strains were only used for quantification and comparison of gross mechanical stiffness.

Specimens were then relaxed and carefully removed from the arms using a razor blade and their central corneal thickness was measured again using a pachymeter.

### X-ray scattering

Small-angle X-ray scattering (SAXS) were carried out on beamline I22 at Diamond Light Source, the United Kingdom synchrotron (Smith et al., 2021). The beam wavelength was 1 Å, with an elliptical profile approximately 250 µm by 150 µm with major axis parallel to the direction of load. A Pilatus P3-2M photodetector (Dectris, Switzerland) collected scattered light at a distance of 5.95 m from the specimen. This arrangement allowed visualisation of scattering features corresponding to fibril-scale architecture (10 nm–100 nm). The 58.38 Å peak associated with the [001] crystal plane reflection of powdered silver behenate was used to centre and calibrate the SAXS images.

**FIGURE 1:**
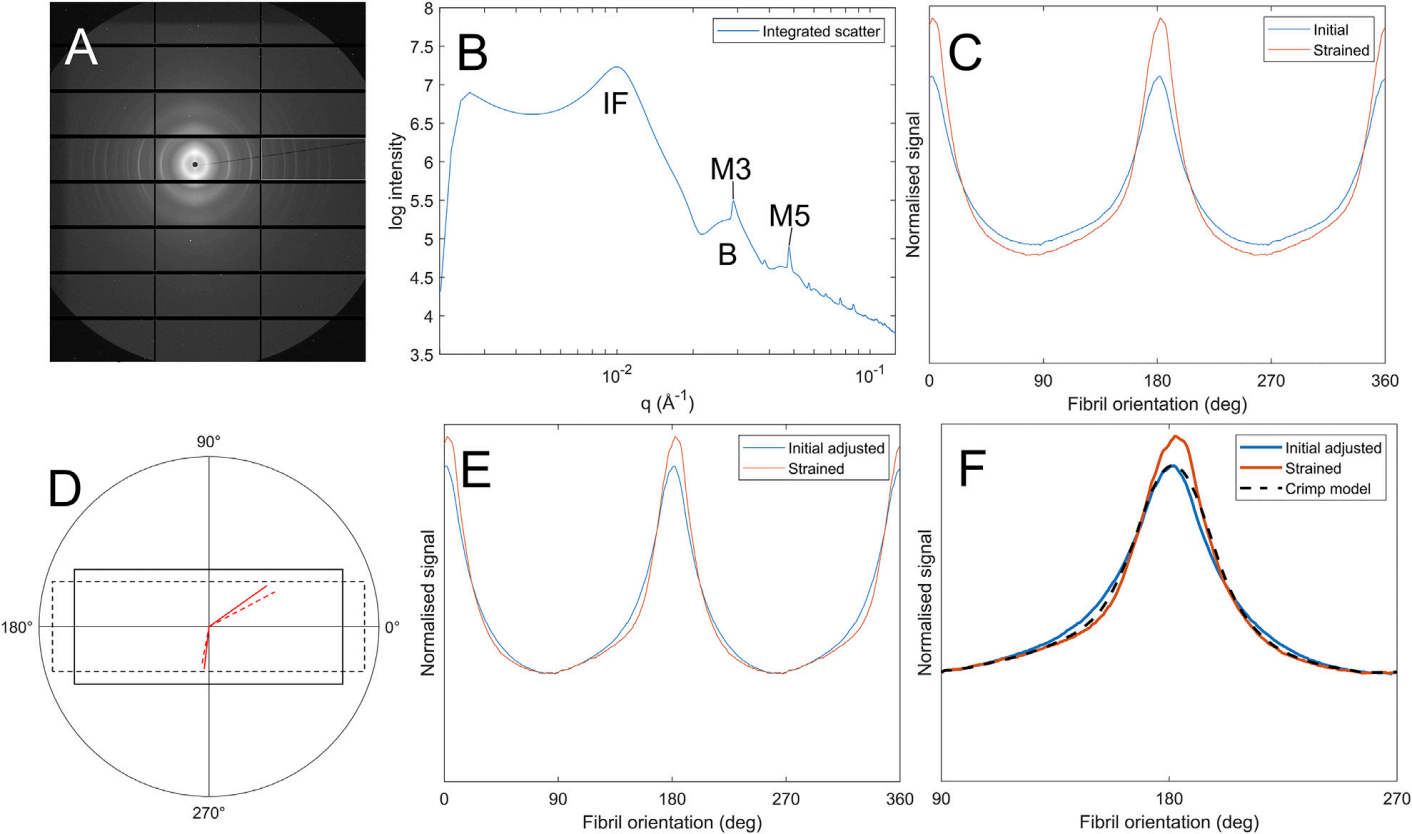
Process of calculating fibril crimp. **(A)** SAXS pattern of corneal collagen. **(B)** Azimuthal integration showing peaks corresponding to the interfibrillar spacing (IF), meridional D-spacing (M3, M5) and the Bessel-shaped cylinder transform (B) **(C)** Polar dependence of interfibrillar diffraction peak, $F\left(\chi \right)$ , for a sample in its initial state and when subjected to tensile strain. **(D)** Illustration of how affine deformations affect fibril orientation (solid lines are the initial geometry, dashed lines are the deformed geometry). **(E)** Polar data from panel C with the initial distribution adjusted to compensate for the affine deformation of the strained state, this distribution is referred to as $F_{c}\left(\chi \right)$ . **(F)** The same polar data as in panel E with the crimp model overlaid, which calculates the crimping required to map the strained dataset to the initial adjusted dataset.

SAXS images were acquired from a region approximately 1.5 mm × 1.5 mm about the centre of each specimen by controlling the beamline stage in conjunction with the tensile tester. This avoided sample edges and associated cutting artefacts (Bell et al., 2022), ensured strains were largely uniaxial, and minimized intraspecimen variability in fibril structure (it has been shown elsewhere the porcine central cornea fibril arrangement is largely invariant with lateral position (Hayes et al., 2007)). An example SAXS pattern from the centre of a pig cornea is shown in Figure 1A. Analysis of scatter patterns to quantify the hierarchical structure of corneal collagen has been described elsewhere (Bell et al., 2018). Briefly, a data reduction process (Pauw et al., 2017) was run to remove beamline artefacts from the data, such as dead pixels, panel edges, beam flux fluctuations, etc., and then a circumferential integration was carried out to produce an intensity (I) vs. wavevector (Q) dataset**.** The Porod background, which takes the form I(Q) ∼ Q^−4^ **,** was removed from peaks of interest through an automated, iterative process whereby a linear polynomial was fitted to a plot of log(I) vs. log(q) (Figure 1B) such that the area under the fit was minimised. Features of interest in this study are labelled in Figure 1B. The interfibrillar Bragg peak (IF) allows measure of the lateral spacing of fibrils, the meridional peaks (M3, M5) are diffractive orders of the D-period and the Bessel-shaped cylinder transform peaks (B) provide a measure of the fibril diameter. The azimuthal dependence (commonly referred to as χ ) of the IF peak was used as a measure of the fibril orientation distribution, such as in Figures 1C,E.

Hydration was approximated through measures of specimen thickness and observation of interfibrillar spacing, trends in which have been investigated previously (Hayes et al., 2017). Measures of specimen thickness were made before and after imaging using a pachymeter, although this is quite imprecise on tensile strips (variability of ∼5%). It was not possible to use weight as a measure of hydration, as the use of cyanoacrylate glue precluded like-for-like weighing before and after imaging. Exclusion criteria were: (i) Interfibrillar spacing outside of range shown in (Hayes et al., 2017) (at H ratios of 2.9–3.4, IFS^2^ range is approximately 500 mm^2^, so using the fit parameters provided this equates to an IFS range of approximately ±6 nm about the mean); (ii) Measured thickness drop in excess of 10%. No samples in this study dried to the extent that data needed to be excluded and measurements pertaining to hydration are included as a supplement.

### Hierarchical strain characterisation

The reorientation of fibrous structures under external load is a nonlinear process that is dependent on several factors, including changes in gross geometry of the tissue, extracellular matrix interconnectivity, fibril waviness/crimp and the ability of the crimp to straighten. To approximate and separate the different strain mechanisms, the analysis was split into three stages.

#### Fibril elongation

Fibrils can elongate via several mechanisms, but under small loads we have shown previously that the dominant mechanism is the *spring-like* straightening of supramolecular twist (Bell et al., 2022). The straightening of “tropocollagen springs” gives rise to a change in D-period (referred to as “D-strain”), so precise measurement of the relative changes in the M3 peak provides a measure of fibril elongation that is separate from other strain mechanisms. In contrast to most approaches to SAXS analysis, the mean peak value was chosen as opposed to the peak (statistical mode) to provide a measure of mean fibril elongation, i.e.

$$\varepsilon_{n,fibril}=\frac{D_{n,mean}}{D_{0,mean}}$$

where $\varepsilon_{n,fibril}$ is the fibril-scale strain at the *n*th data point, $D_{0,mean}$ is the mean value of the D-period before load and $D_{n,mean}$ is the mean value of the D-period for the *n*th data point. The mean was used in preference to the mode in order to make meaningful statistical comparisons between groups and strain values.

#### Affine deformation

Assuming sinusoidal crimp (Jan et al., 2018), the orientation distribution of fibrils $F\left(\chi \right)$ , obtained from the azimuthal dependence of the IF peak is a convolution of the sinusoid describing the crimp and the orientation distribution of the crimped fibrils (see Bell et al. (Bell et al., 2022) for a detailed explanation). In the absence of a separate imaging technique such as digital image correlation to quantify the local strain, the fibril orientation density (the number of fibrils aligned within a particular orientation range) around the direction orthogonal to the applied strain can be used to approximate affine sample deformations (in this case, the amount of stretch parallel to the applied load, and the amount of contraction perpendicular to it, close to where the data is collected). This relies on the assumption that changes in fibril orientation associated with strain-induced uncrimping only affect fibrils aligned within some angular range of the applied load (fibrils aligned outside of this range will not be under tension and not be uncrimping). Figure 1C shows typical fibril orientation data for a specimen in a strained and unstrained state, with a clear reduction in the amount of collagen aligned orthogonally to the applied load (load direction 0° and 180°). Affine changes are schematically illustrated in Figure 1D–assuming the strain is invariant with position, the solid lines represent the unstrained geometry and the dashed lines represent the strained geometry. The affine strain causes a reorientation in the angled lines. This reorientation is calculated for each 0.5° increment that the initial fibril orientation was sampled over, for a range of plausible affine deformations (strains parallel and perpendicular to the applied load that the corneal strip could be expected to experience) until a fit is reached with the strained data. The reason the number of fibrils in the orthogonal direction decreases with an affine deformation is because, for a given range of initial orientations, the reorientated range is wider–e.g., a fibril aligned at 90° will stay at 90°, while a fibril at 91° will reorientate to approximately 91.1°. Hence, fibrils initially aligned between 90° and 91° now occupy a range of 90°–91.1° and the fibril orientation density reduces. This effect is reversed close to the direction of applied load. The resulting affine-compensated distribution $F_{c}\left(\chi \right)$ is shown in Figure 1E, and with affine effects accounted for the remaining difference between the initial adjusted distribution and the strained distribution is assumed to be associated with uncrimping.

#### Uncrimping

Calculating directly the effect of uncrimping on an orientation distribution is a challenging problem, however the inverse problem (applying crimp to a dataset) simply involves convolving the distribution with that of a sinusoid (see (Bell et al., 2022) for more details). Firstly, the orientation distribution F_c(χ) with least crimp was assumed to be that with the largest slenderness ratio (the ratio of height to width of the orientation peak). The difference in crimp states between the least crimped distribution and another distribution can then be approximated by convolving the strained dataset with sinusoids of the form y=A⁡sin⁡θ where *A* is iterated until the best fit with the unstrained data is found. This is computationally quick, so a brute force approach to find *A* is feasible. The value of *A* that maps the least crimped distribution to each other distribution (see Figure 1F) for a given specimen is then taken as the crimp state. Note that this is a measure of *relative* crimp, as it is not possible to know how crimped the least crimped state was to obtain an absolute value. This also means that the ability to compare specimens is limited as the least crimped states for two individual specimens are unlikely to be the same. We therefore limit comparisons of crimp data to averages between treatment groups.

## Statistics

This study explores the effect of CXL and decorin treatment (as individual treatments and as a combined treatment) on biomechanical and structural metrics. To quantify the effect of CXL we compared groups CXL and Dec + CXL with Ribo and Dec, whereas to quantify the effects of decorin application we compared Dec and Dec + CXL groups with Ribo and CXL. T-tests were used to assess significance with the null-hypothesis rejected at the 0.05 confidence level.

To quantify correlations between metrics, the Spearman’s Rho correlation was used with the null-hypothesis rejected at the 0.05 confidence level.

## Results

### Tensile stiffness

**FIGURE 2:**
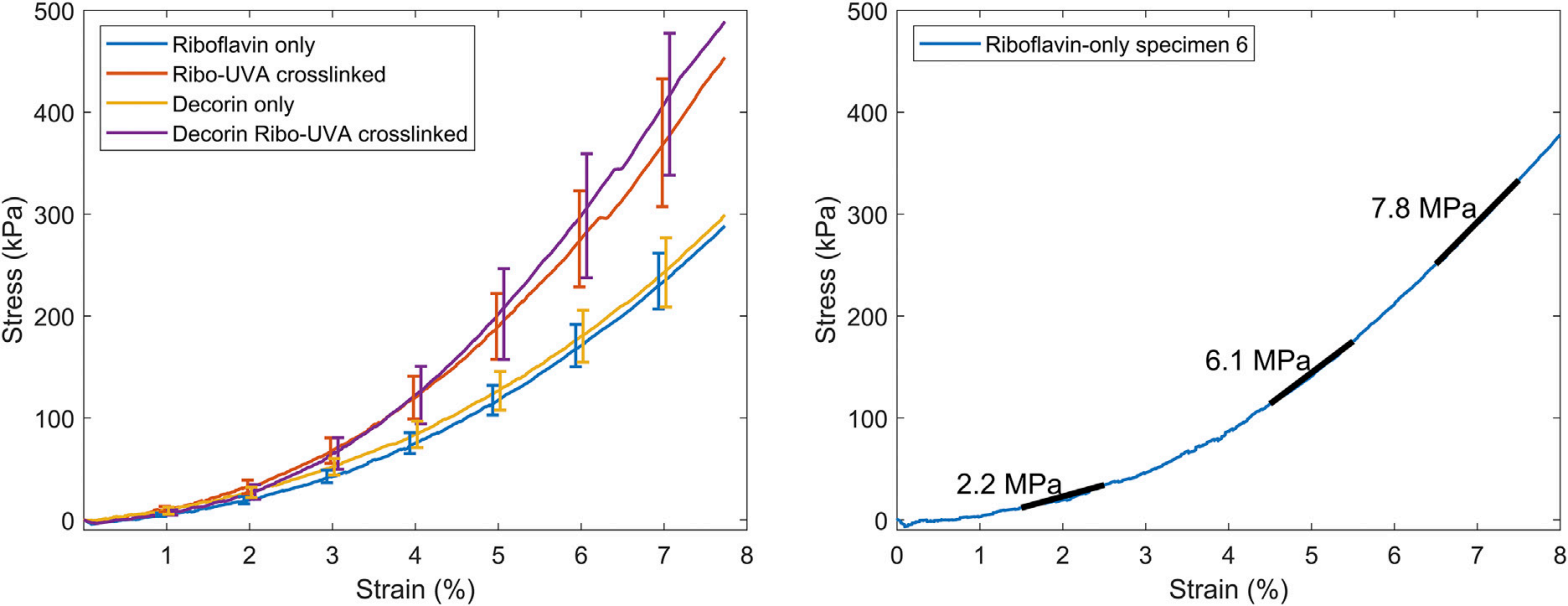
Left. Averaged stress-strain plots for each group (bars SEM). Right. Representative stress-strain plot from a riboflavin-only treated corneal strip with the tangent moduli overlaid.

**FIGURE 3:**
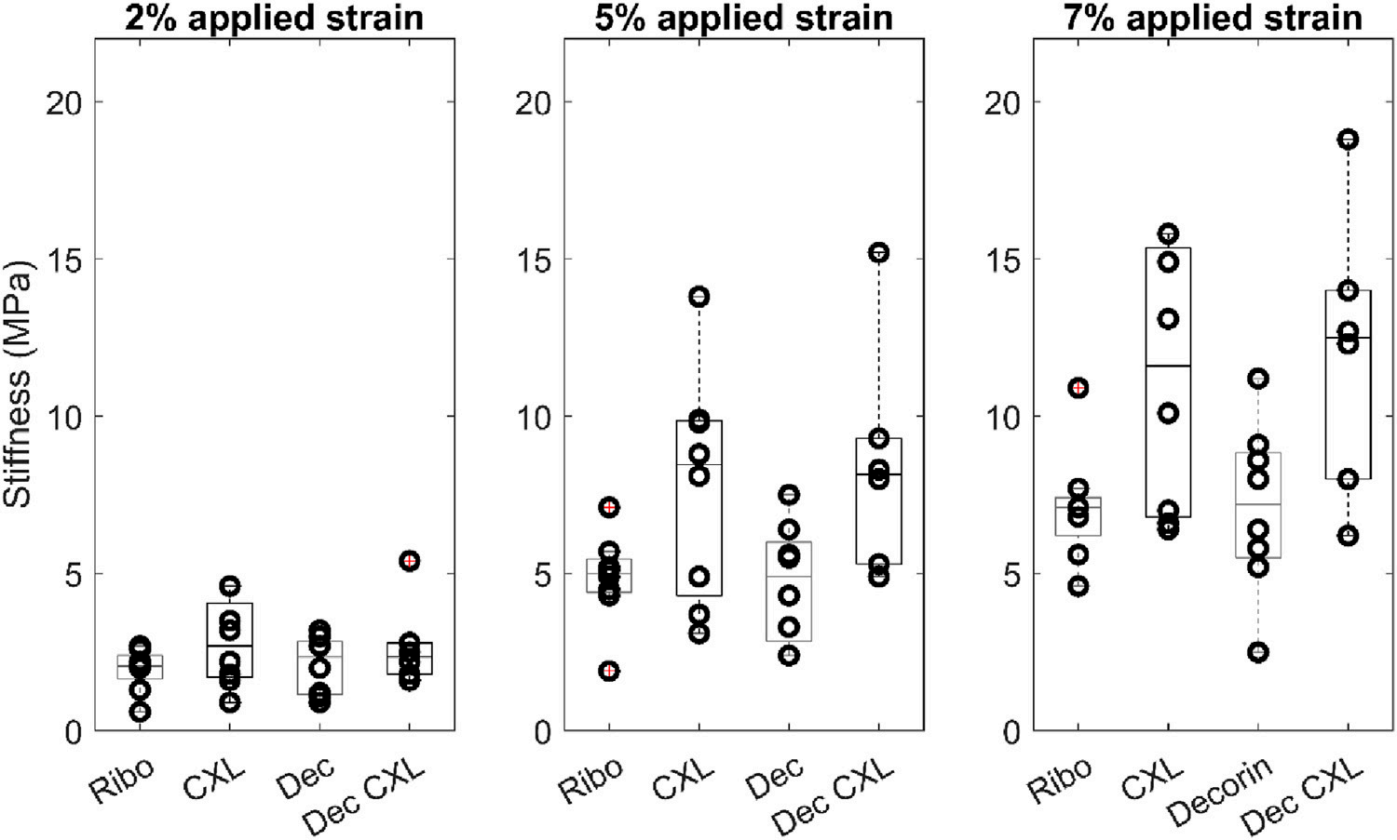
Tangent moduli for each group at 2%, 5% and 7% applied strain. Boxes show interquartile range and mean.

Figure 2 shows averaged stress-strain plots for each treatment group alongside representative data from a single riboflavin-only treated specimen with tangent moduli calculations at 2%, 5% and 7% applied strains. The calculated tangent moduli for each treatment group at 2%, 5% and 7% applied strain are shown in Figure 3 and Table 1.

**TABLE 1:** Average tangent stiffness (in MPa) of each group as a function of applied strain.

Applied strain	2%	5%	7%
Ribo	1.8 ± 0.3	4.6 ± 0.5	6.7 ± 0.7
CXL	2.8 ± 0.5	7.8 ± 1.3	11.2 ± 1.5
Dec	2.1 ± 0.3	4.7 ± 0.7	7.1 ± 1.0
Dec + CXL	2.7 ± 0.6	8.5 ± 1.5	12.0 ± 1.8

CXL, and Dec + CXL, groups were significantly stiffer than Ribo and Dec groups, respectively at applied strains of 2% (p < 0.05), 5% (p < 0.01) and 7% (p < 0.01). Decorin applied in isolation or with CXL, did not affect stiffness significantly.

### Fibril elongation/D-strain

**FIGURE 4:**
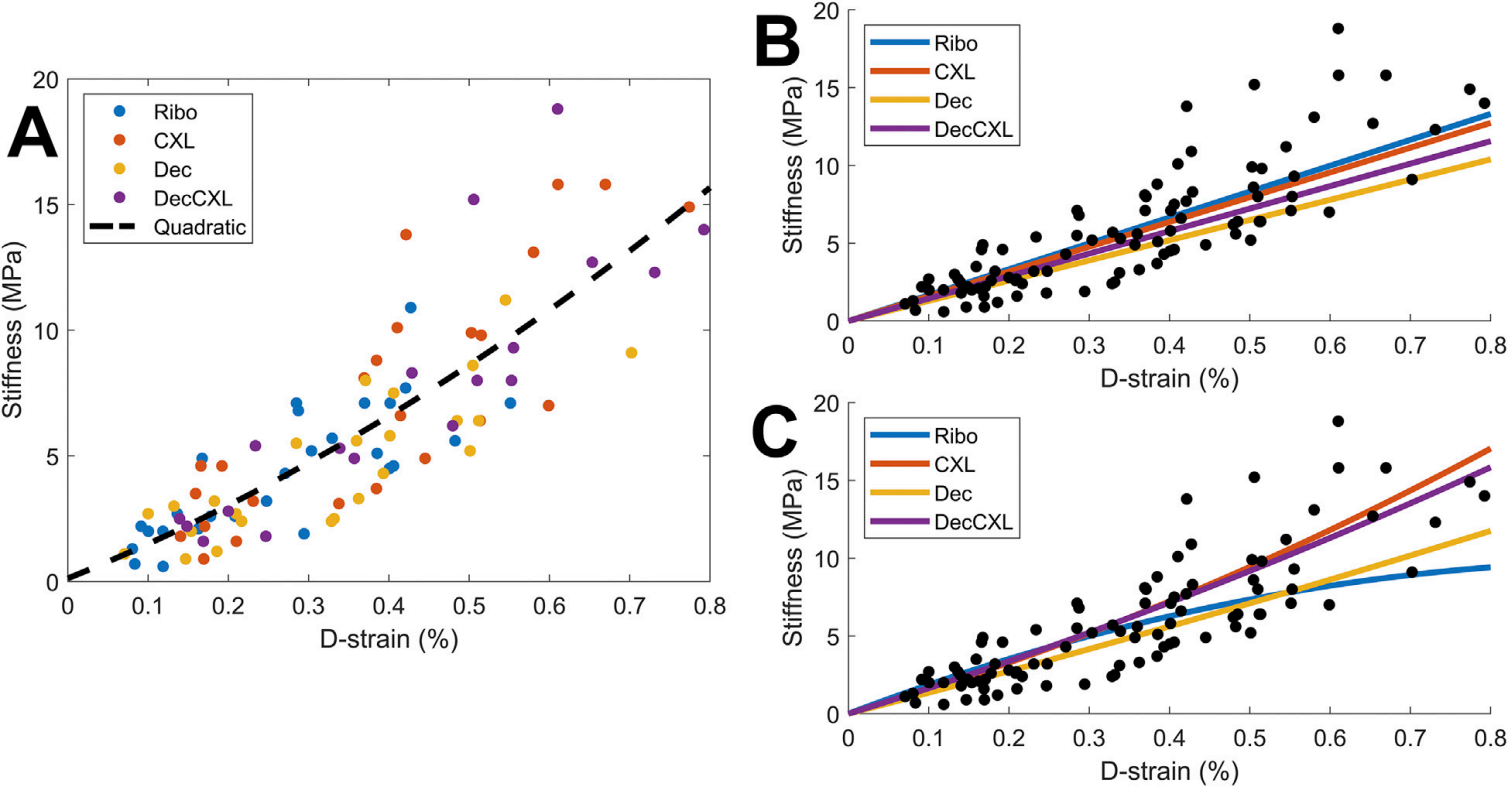
Comparison of D-strain and associated tangent stiffness for all samples at 2%, 5% and 7% applied strain. The distribution appeared nonlinear, so different fits were tested (see text for details). **(A)** Comparison of individual values with an overall average quadratic fit. **(B)** Linear fits for each group based on data up to D-strain of 0.4%. **(C)** Quadratic fits for each group.

D-strain was found to correlate with stiffness when all specimens were combined (2%: p < 0.05, 5% and 7%: p < 0.0001)**.** To assess whether either CXL or decorin treatment stiffens fibrils individually, we explored the relationship between D-strain and tangent stiffness, and a scatter plot comparing the values between each group is shown in Figure 4A. The overall trend is approximately linear for all groups up to 0.4% D-strain, and for D-strains higher than this (data for which is dominated by the CXL and Dec + CXL groups) the stiffness increases more nonlinearly. To make quantitative comparisons of fibril stiffness between treatment groups, a reference trendline needed to be established. Linear fits through the origin for data up to a D-strain of 0.4% are shown in Figure 4B (gradients: Ribo: 1.66 GPa, CXL: 1.59 GPa, Dec: 1.30 GPa, Dec + CXL: 1.44 GPa), however these were particularly sensitive to noisy low-strain data and it has been suggested previously that the relationship between D-strain and fibril stiffness is nonlinear (Leighton et al., 2021). By comparing the average fractional difference between each group’s datapoints and the quadratic trendline in Figure 4A, we can approximate the fibril stiffness relative to the average over all groups (Ribo: +4%, CXL: +8%, Dec -11% Dec + CXL: +2%). This showed that fibrils in the decorin treated group were significantly less stiff than those of the other three groups (p < 0.05). While the quadratic trendline belies what is likely a much more complex relationship between D-strain and stiffness, it provides a measure of differences between groups, which are small compared to their differences in stiffnesses. This implies that fibril stiffening is a relatively minor contributor to the overall tissue stiffening effect associated with CXL. Due to the relatively small group size and large inter-sample variability, quadratic fits for each group (Figure 4C) were not a useful comparison.

The uniformity of D-strain was investigated by quantifying the slenderness of M5 and its change with strain for fibrils aligned close to parallel to the applied load. This effectively measures the range of strains being experienced by fibrils, with a higher uniformity meaning a smaller range of strains. Slenderness was quantified by measuring the height of the peak and dividing it by the full width at half maximum (FWHM), which effectively measures the variance of fibril D-period within the scanned volume. Decorin treated datasets (Dec and Dec + CXL) were significantly more uniform than Ribo and CXL at 2% and 7% applied strain (p < 0.01) (Table 2).

**TABLE 2:** Structural metrics and their change with applied strain.

	D-strain (%)		D-strain uniformity (a.u.)		Fibril reorientation (%)		Fibril uncrimping (a.u.)	
Applied strain	2%	7%	2%	7%	2%	7%	2%	7%
Ribo	0.12 ± 0.03	0.40 ± 0.09	6.53 ± 0.43	6.46 ± 0.46	0.4 ± 0.5	1.3 ± 1.7	6.9 ± 7.3	0.4 ± 5.7
CXL	0.18 ± 0.03	0.57 ± 0.12	6.85 ± 0.50	6.97 ± 0.37	1.4 ± 0.8	4.1 ± 1.6	12.4 ± 9.9	7.4 ± 2.5
Dec	0.15 ± 0.05	0.48 ± 0.12	7.14 ± 0.70	7.14 ± 0.70	0.9 ± 0.9	2.2 ± 1.3	5.5 ± 5.0	−3.1 ± 2.5
Dec + CXL	0.19 ± 0.04	0.63 ± 0.12	7.52 ± 0.91	7.53 ± 0.91	1.4 ± 0.9	4.2 ± 2.6	15.5 ± 6.2	9.8 ± 2.1

### Fibril reorientation

The fibril orientation distribution, $F\left(\chi \right)$ , (an example of which is shown in Figure 1C), can be integrated to determine the relative orientation density between different angular ranges. The amount of collagen aligned within ±45° of the direction of applied load was compared with that aligned within ±45° of the perpendicular direction. At rest the ratio of these values:

$$\frac{\int_{-45}^{45}F\left(\chi \right)}{\int_{45}^{135}F\left(\chi \right)}$$

was approximately 60:40 in favour of the parallel orientation range for all samples. The ratio generally increased as strain increased, however the increase was significantly higher for the Ribo/UVA crosslinked specimens than non-crosslinked specimens (see Table 2; confidences: 2%: p < 0.01, 7%: p < 0.001). It was found that the extent of parallel:perpendicular reorientation correlated strongly with D-strain and stiffness when all groups were combined (2%: p < 0.01, 7%: p < 0.001).

### Crimp

**FIGURE 5:**
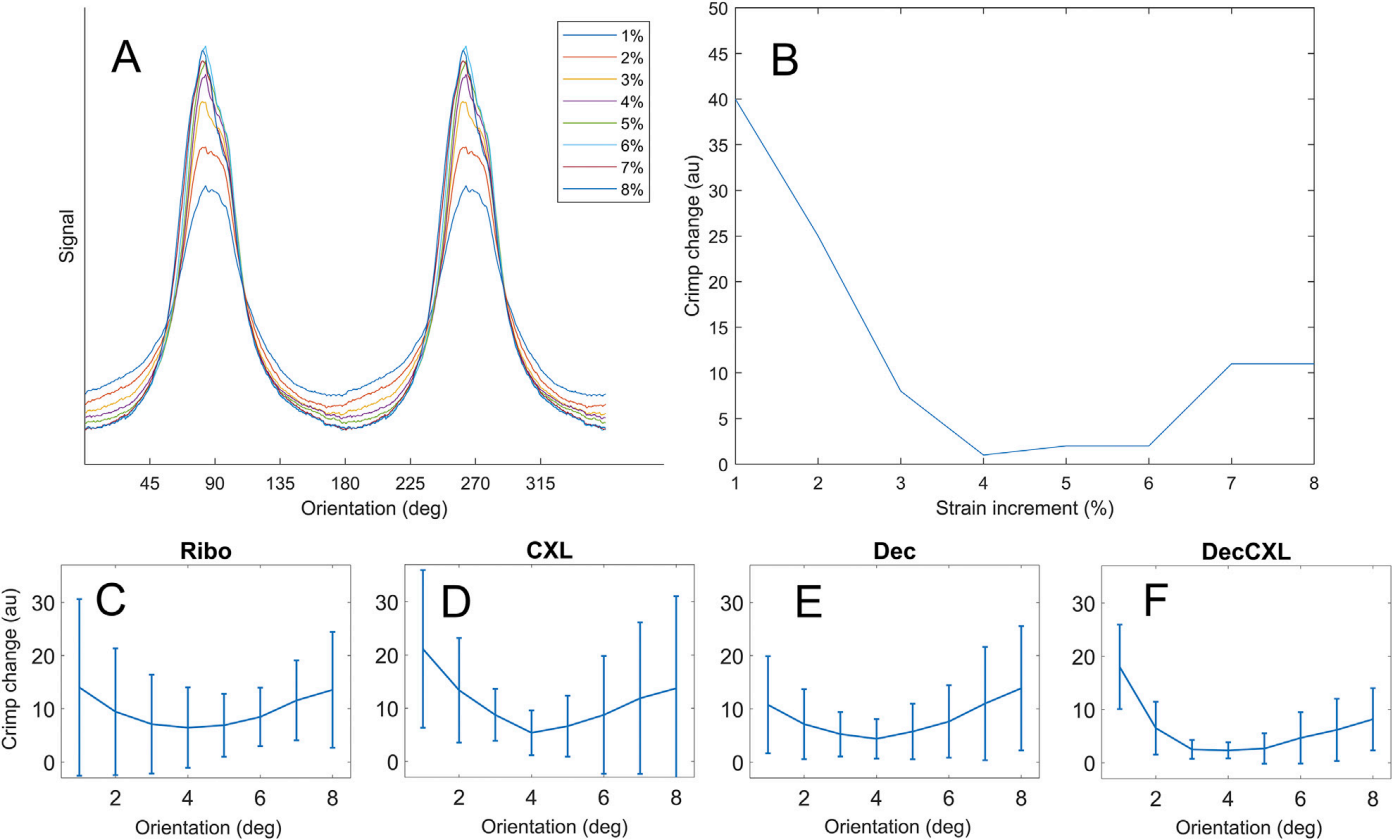
Crimp data. **(A)** Polar arrangement of fibrils for a representative specimen at each strain increment. **(B)** Extent of crimp at each strain increment relative to the least crimped state. **(C–F)** Averaged crimp extents for each group (bars mean ± SD).

Fibril crimp was estimated by obtaining the affine-compensated orientation distribution $F_{c}\left(\chi \right)$ when fibrils were estimated to be straightest under applied load (chosen as the distribution with largest slenderness ratio–the ratio of height to width of the peak). This distribution was convolved with sinusoids of varying tortuosity, adjusted by varying their amplitude *A*, until a fit with $F_{c}\left(\chi \right)$ at other strains was found. *A* was taken as a relative measure of crimp with strain for each sample. CXL groups exhibited significantly different uncrimping trends to non-CXL groups at 2% (p < 0.05) but not at 7% (Table 2). It was found that the modelled crimp for most samples initially trended down before increasing again at higher strains (see Figure 5). The upward turn in crimp for each sample corresponded with a reduction in slenderness of the polar distribution of fibrils, a change in the trend of movement of the mean orientation and often the appearance of a shoulder in the distribution. This suggests that the assumption that fibril reorientation is simply a convolution of affine deformation and uncrimping breaks down somewhere between 2% and 7% applied strain, with nonlinear effects becoming significant. Given these nonlinear effects, while we report the results of the crimp model for all strains to illustrate the more complex rearrangements of corneal collagen, we suggest that the model only isolates uncrimping behaviour at the 2% data point. At this datapoint, D-strain and crimp correlated (p < 0.05) when all groups were combined.

## Discussion

The primary aim of this study was to characterise the structural changes that occur in riboflavin/UVA crosslinking (CXL) that give rise to enhanced biomechanical properties. We also investigated the structural and biomechanical impact of applying human recombinant decorin core protein to the corneal stroma, in isolation and in combination with CXL. CXL resulted in a significant increase in the tangent stiffness of corneal strips at all applied strains (2%–7%). The increase in tangent tensile stiffness caused by CXL was approximately 60%, which falls between values reported in the literature (Hammer et al., 2014; Wollensak and Spoerl, 2004; Chang et al., 2018). However, contrary to previous reports (Pappa et al., 2021), we found that a 60 s application of decorin core protein to the de-epithelialised corneal stroma did not significantly increase the stiffness of the cornea. Similarly, when decorin core protein application was combined with CXL (Dec + CXL), the tissue stiffening effect did not extend beyond that achieved with CXL alone.

To elucidate the mechanism underpinning CXL stiffening, X-ray scattering was employed to probe the microstructural mechanical environment. Our attention focused on fibrillar collagen, as the most abundant loadbearing structure in the cornea, specifically how fibrils elongate and reorientate. We quantified fibril elongation through close inspection of meridional diffraction peaks associated with the D-staggered structure of the collagen fibril. The increase in D-period associated with the application of tensile strain was found to correlate very significantly with tissue stiffness when all treatment groups were pooled together. Furthermore, factoring in the nonlinear relationship between D-strain and specimen stiffness, our data suggests that stiffening of individual fibrils is likely only a minor contributor to the overall increase in tissue stiffness following CXL. This finding is not entirely surprising, based on our previous studies which have indicated that intra-fibrillar cross-linking may be limited to just the collagen molecules at the surface of the fibrils. However, it should be noted that whilst CXL is limited to the anterior 300 microns of the cornea, the X-ray scatter patterns represent average measurements of collagen parameters throughout the entire thickness of the cornea. As such, detected changes in collagen parameters reported in this study should be viewed as conservative estimates of treatment effects.

Assuming minimal stiffening occurs within individual collagen fibrils then the origin of CXL stiffening must be a change in larger-scale strain mechanisms, and the data overall shows clearly that stiffer specimens experience more fibril reorientation. This could be explained by a greater fraction of fibrils being recruited (recruitment meaning fibrils being pulled to an extent that they contribute to the tissue stiffness), and/or a reduction in the strain required to recruit fibrils, and this would effectively stiffen the tissue. These effects could be achieved by the induced cross-links providing stronger and more plentiful links between extracellular matrix proteins, resulting in a form of interfibrillar bracing (linking together of fibrils such that they share loads more evenly). The patterns in fibril reorientation support this hypothesis, as there was significantly more in CXL specimens than non-CXL specimens. The patterns in strain uniformity also somewhat support this hypothesis–decorin treated samples exhibited significantly greater strain uniformity (i.e., reduced variance in D-strain) than non-treated samples, although CXL samples did not (mean strain uniformity increased but not significantly, p = 0.1). It is possible that this is a real effect in CXL that is masked by only treating approximately half of the tissue thickness (Caporossi et al., 2006) while SAXS data is averaged through the full thickness of the specimen. A clear answer to this will require greater statistical power or the analysis of entirely treated tissue. It is not currently known how deep decorin solution penetrates into the tissue. It should be noted here that riboflavin/UVA-induced crosslinks cannot themselves bridge between and brace fibrils, but they can reinforce structures that do, as we discuss later.

When fibrils are braced together more, their ability to straighten is likely to be impacted. This is because the periodicity of crimp in corneal collagen is in excess of 10 µm (Jan et al., 2018) while the periodicity of binding sites for crosslinks to form must be at most 67 nm (the length of a D-repeat). The complex, multidirectional movement involved in uncrimping will therefore tug at other fibrils, forcing them to move in unison. Our modelling of crimp revealed that CXL specimens exhibited significantly more uncrimping than non-crosslinked specimens, supporting the hypothesis that more fibrils are engaged in treated tissues. This conclusion is derived from the fact that fibrils require more force to straighten as they become less crimped, meaning the output of the model for a small fraction of fibrils straightening due to a fixed load will be less than a larger fraction of fibrils straightening under the same load. This was, however, limited to the 2% datapoint as at greater applied strains the modelling was complicated by changes in the shape of the orientation distribution. It was noted qualitatively that CXL specimens deviated more from the typical smooth bimodal distribution (Figure 5A) under strain than non-CXL specimens (such as through the formation of shoulders and additional mini-peaks).

**FIGURE 6:**
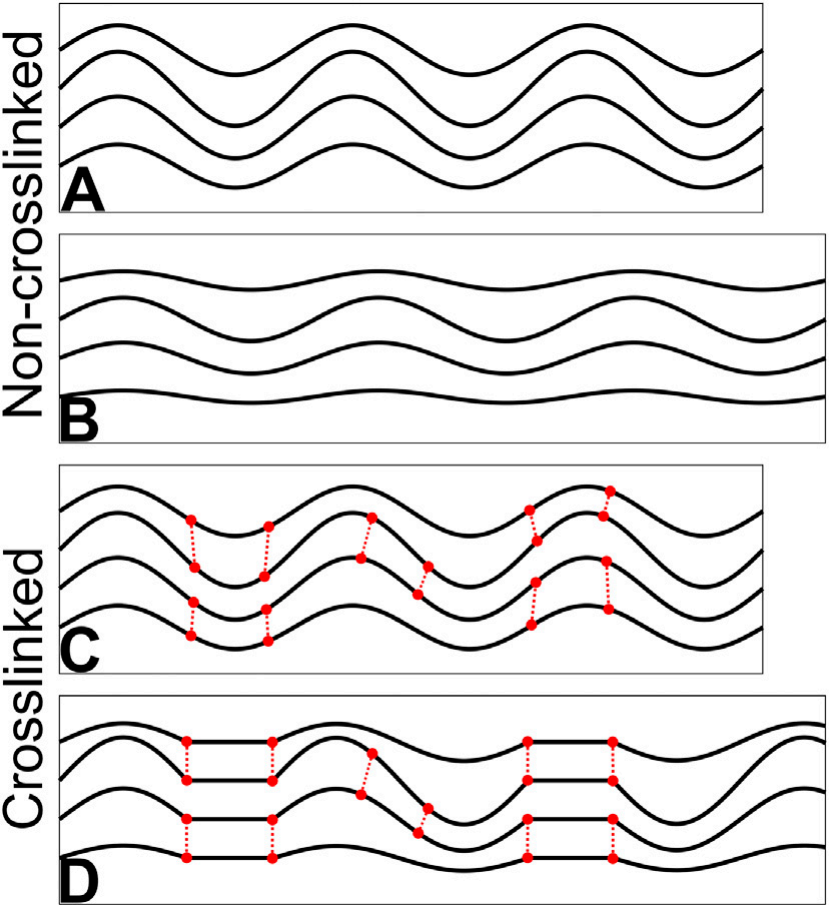
Illustration of uncrimping and the effect of crosslinking. **(A)** Crimped fibrils in an unstrained state. **(B)** When strained, some fibrils straighten and recruit before others; here the top and bottom fibrils are contributing more to specimen stiffness than the middle two. **(C)** The same arrangement as A with crosslinks between fibrils. **(D)** When strained, the top and bottom fibrils recruit along with the connected regions of the middle fibrils. A greater proportion of fibrillar collagen resists the tensile load in D than B, making it stiffer.

**FIGURE 7:**
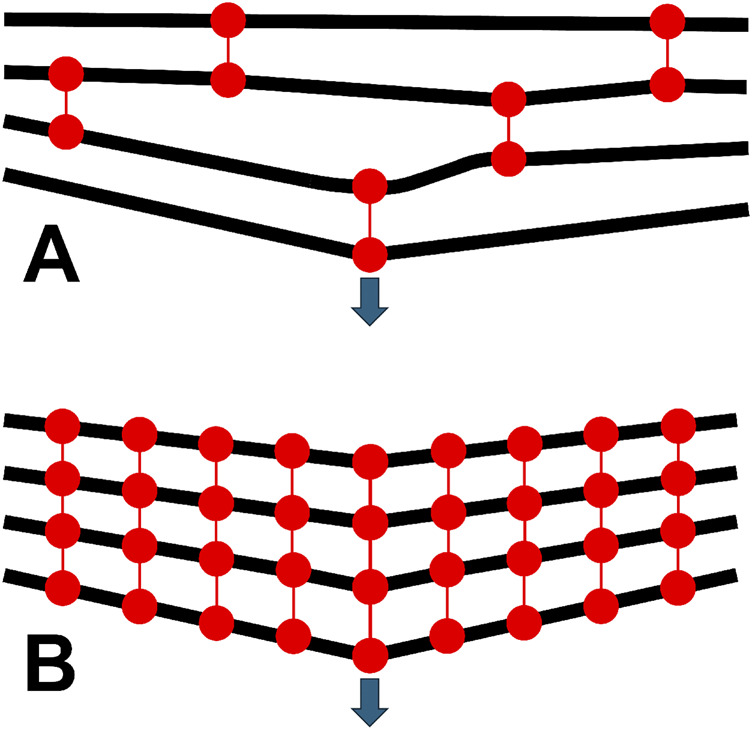
Illustration of the reorientation behaviour of collagen fibrils (black lines) in **(A)** untreated tissue and **(B)** crosslinked tissue. The resting configuration of the fibrils is horizontal and equally spaced. The crosslinked tissue exhibits more total fibril-scale reorientation when a point strain is applied (arrow).

While fibrils are probably too interconnected to uncrimp independently even without exogenous crosslinking, any added interconnectivity is likely to have two effects. Firstly, load will be distributed more evenly across fibrils in the tissue. Where in the untreated state the mechanical response is dictated by the rate of fibril recruitment, the interconnected nature of treated specimens means more tortuous (wavy) fibrils will have a greater contribution. This is supported by the increase in D-strain in CXL specimens. An illustration of this is shown in Figure 6. This is somewhat analogous to how tight-woven fabrics are stiffer than those with a loose weave; a tight weave means load is shared over more material with less ability for strands to straighten or slip independently. The second effect relates to fibril reorientation, which will be more sensitive to strain as the shorter lengths between braces will require smaller deflections to achieve the same reorientation. As the number of braces between fibrils increases in density, the response to exogenous strain will approach that of a continuum (illustrated in Figure 7). Continuing with the woven fabric analogy, this is similar to adding extra stitches to a loose weave, holding strands in close conformity so pulling or pushing of a region of the fabric causes all the strands in the vicinity to reorientate, not just the ones directly loaded.

Corneal collagen structure is mediated by a network of interconnected glycosaminoglycans (GAGs), which bind to adjacent collagen fibrils via decorin and lumican proteoglycan core proteins (Scott and Haigh, 1988; Meek et al., 1986). It has been shown that CXL forms crosslinks between fibrillar collagen and decorin (Zhang et al., 2011), which envelopes tropocollagen molecules in a horseshoe-shaped structure and enhances the stiffness of type 1 collagen gels (Reese et al., 2013). However, the binding force between collagen amino acids and decorin core proteins has been modelled as being greater than the ultimate strength of GAG chains, suggesting enhanced collagen-proteoglycan binding may not contribute significantly to extracellular matrix stiffness (Vesentini et al., 2005). The creation of reactive oxygen species during the CXL process is likely to alter the chemistry of some GAGs, although it has been noted that sulphation has an insulating effect on GAG depolymerisation *in vitro* (Moseley et al., 1995). The mediating effect of minor collagens, which are abundant in cornea (Zimmermann et al., 1986), and their potential to act as braces themselves, has yet to be explored.

There were several notable challenges and limitations associated with this study. Firstly, due to the techniques employed and the quantity of tissue required, it was necessary for the study to be performed using post-mortem porcine eyes. Post-mortem corneas are subject to posthumous swelling, and changes in tissue hydration are well known to affect the ultrastructure (Hayes et al., 2017) and biomechanical properties of the tissue (Elliott and Hodson, 1998)**.** To minimise the impact of such changes, tissue was obtained and treated within 6 h of death and corneal thickness measurements, made at regular intervals throughout the treatments and prior to data collection, were used to monitor levels of tissue hydration. Furthermore, the order of treatment and data collection (alternating eyes from each group) and the use of a non-irradiated, riboflavin-only control group (in preference to untreated corneas) helped ensure that there was no significant difference in corneal thickness/hydration between the groups. Comparisons of absolute tensile stiffness values to others in the literature should be made with caution, as the slenderness ratio (ratio of length to width) of specimens in this study was approximately 3:1. While this meets the minimum standard for testing polymer matrix composite materials (ASTM Standard, 2008) it does not meet the recommended values (which need to be especially strict for anisotropic materials (Legerlotz et al., 2010)), meaning the stress environment in the strip will comprise both uniaxial and planar components. This is an unavoidable issue associated with the size of the cornea and the need to mitigate the effects of cut edges and explains in part the variation in stiffness results across studies in the literature. It should also be noted that the nature of the test and the necessary straightening of corneal curvature perturbs specimens from their physiological state, meaning any direct physiological comparisons should be made with caution. The use of synchrotron X-ray scattering, which obtains data through the full thickness of specimens, means results are insensitive to depth-dependent variation. Follow-up studies using complementary techniques will be of particular value for evaluating changes associated with CXL, which only affects the anterior 300 µm of the tissue.

The results have some interesting implications for both the clinic and wider field of connective tissue research. Firstly, the findings of this study, which indicate that the stiffening effect in CXL treated corneas is primarily due to an increased interconnectivity of the fibrillar collagen network rather than a stiffening of the fibrils themselves, represent a major step forward in our understanding of the mechanism by which CXL therapy stops keratoconus progression, and has important ramifications for the accuracy with which computational models (based on structural data), may predict the outcome of surgical procedures. Furthermore, this is an extracellular example demonstrating how the interconnectivity of biopolymer networks can tune gross mechanical stiffness (Gardel et al., 2004; Head et al., 2003). The results highlight the need to further explore the role of proteoglycan and minor collagens in the crosslinking process, particularly whether their exogenous application or removal could allow for greater control over CXL treatment, and ultimately better outcomes for patients. The findings also provide a new dimension to explore in the mechanical modelling of cornea and soft tissue in general as we begin to incorporate fibril-scale architecture.

In conclusion, we have shown that Riboflavin/UVA crosslinking enhances the tensile stiffness of corneas by approximately 60%, while decorin treatment does not change the stiffness significantly. The stiffening effect appears to be associated with increased interconnectivity of the fibrillar collagen network as opposed to stiffening of the fibrils themselves, although a small increase in fibril stiffness was measured. While decorin treatment did not affect the macroscopic mechanical properties, it did enhance the interconnectivity of fibrillar collagen whilst also reducing fibril stiffness. Combining decorin treatment with Riboflavin/UVA crosslinking did not significantly enhance the stiffness over crosslinking alone, and as such is unlikely to be useful in the treatment of keratoconus. The methodology developed here can be adapted to a broad range of tissues and conditions for the evaluation of new and adjuvant therapies.

## Data Availability

All data supporting this study is openly available in Figshare data repository at 10.17035/cardiff.30122182.
