# Supplementary material for: Hierarchical biomechanical characterisation of riboflavin-UVA crosslinking and decorin treatment in the porcine cornea
Source: Front Bioeng Biotechnol. 2025 Sep 24;13:1603679. doi: 10.3389/fbioe.2025.1603679 (PMC12504365; doi:10.3389/fbioe.2025.1603679)
Supplement: Supplementary file 1 [file DataSheet1.pdf]

Supplemental material

Hydration measurements

Pachymetry was used to measure central corneal thickness before and after data acquisition, with a reduction in thickness attributed to a drop in hydration. Owing to the imprecise nature of pachymetry on tensile strips (SD of repeat readings approximately 5%), D-period and interfibrillar spacing were used as verification of hydration data. D-period was used as a check of significant dehydration during the experiment, as the shape and position of meridional peaks change significantly when collagenous tissue dehydrates (this did not happen in this study). Interfibrillar spacing was used as a post-experiment verification and was based on analysis in [34].

Hydration can be quantified as follows:

$$H = \frac{wet\ weight - dry\ weight}{dry\ weight}$$

and physiological hydration for the porcine cornea is reported as being approximately $H = 3.2$ with a range of $H = 2.9 - 3.4$ being regarded as "near physiological". According to [34], one would expect the interfibrillar spacing of porcine corneas in the physiological hydration range to vary from the mean by approximately ±6 nm.

For a specimen to be excluded on the grounds of dehydration or overhydration, it needed to either be more than 10% (2SD) different in central cornea thickness when compared before and after the experiment, or more than 6% different from the mean interfibrillar spacing at the strain increment measured. Table S1 summarises the central corneal thickness and IF spacing data (IF spacing data limited to the last strain increment for brevity, which showed the most variation).

Group	Sample number	Thickness before (µm)	Thickness after (µm)	IF spacing (nm)
Ribo	1	710	674	65.15
Ribo	2	682	689	63.17
Ribo	3	675	620	64.56
Ribo	4	671	669	64.29
Ribo	5	735	696	63.43
Ribo	6	731	737	65.98
Ribo	7	841	823	64.81
Ribo	8	719	715	64.44
Ribo	9	776	789	66.35
CXL	1	646	709	68.40
CXL	2	701	636	63.11
CXL	3	659	680	63.89
CXL	4	689	677	63.43
CXL	5	686	763	66.04
CXL	6	683	684	63.36
CXL	7	629	606	69.18

CXL	8	753	726	66.61
Dec	1	704	699	63.58
Dec	2	715	696	66.98
Dec	3	768	782	67.66
Dec	4	659	594	64.51
Dec	5	699	679	67.02
Dec	6	689	677	72.18
Dec	7	659	683	65.82
Dec	8	743	761	68.89
DecCXL	1	677	731	66.04
DecCXL	2	732	687	65.79
DecCXL	3	699	677	61.26
DecCXL	4	633	685	64.80
DecCXL	5	701	755	65.88
DecCXL	6	730	685	66.74

Table A1. Measures of central corneal thickness before and after data collection and final measures of interfibrillar (IF) spacing used for assessment of tissue hydration. All specimens fell within the bounds of thickness change <10% and IF spacing difference from mean <6%.
